# A Study on Consumers’ Perceptions of Museum Cultural and Creative Products through Online Textual Reviews: An Example from Palace Museum’s Cultural and Creative Flagship Store

**DOI:** 10.3390/bs13040318

**Published:** 2023-04-07

**Authors:** Huiqian Huang, He Chen, Yihong Zhan

**Affiliations:** National Research Center of Cultural Industries, Central China Normal University, Wuhan 430079, China

**Keywords:** museums’ cultural and creative products, online textual reviews, consumers’ perceptions, cultural features

## Abstract

The digital transformation and innovative development of museums have led consumers to increasingly prefer purchasing museum cultural and creative products through e-commerce platforms. Although this trend shows potential for market growth, the lack of distinct cultural identity and insufficient product differentiation hinder its stable development. Therefore, this study aims to explore consumers’ perceptions on Palace Museum’s cultural and creative products using cultural hierarchy theory. Taking the Palace Museum’s Cultural and Creative Flagship Store on Tmall.com as a case study, the employed evaluation method involves constructing a lexicon of cultural features using Word2vec model and then analyzing online textual reviews to identify these features. Results reveal that among the various cultural features of the products, consumers placed the greatest emphasis on “Materials used” and the least on “Specialty craft”. With regards to the cultural features of inner “intangible” level, consumers tend to have a limited comprehension and familiarity with the cultural heritage and histories behind the products. This study provides suggestions to museum professionals to optimize the use of traditional cultural resources and develop a product development plan.

## 1. Introduction

The 19th Chinese Communist Party Congress report highlights the importance of protecting and utilizing cultural relics and promoting the innovative development of Chinese traditional culture in the new era [1]. The mission and responsibility of the museum have also changed from the traditional stereotypical image of conservatism and education to an indicator of current trends, representing a unique model of popular consumer culture [2,3]. On the one hand, the cultural and creative products of the museum promote the rapid development of the cultural and creative industry. On the other hand, while realizing the transformation and generating income, it has significantly promoted and carried forward traditional Chinese culture [4,5]. However, this has also resulted in an uneven level of development and a lack of depth in the excavation of cultural connotations, leading to a homogeneity and lack of innovation in these products. Additionally, commercialization of cultural resources may result in vulgarization, cultural distortion and loss of cultural meaning [6]. From the perspective of the consumer, the purchasing decision of consumers for such products is primarily influenced by their cultural awareness [7,8], emotional needs [9] and pursuit of social value [10]. The design of cultural features in these products has a direct impact on consumer choice. Hence, it is vital to conduct research and develop cultural and creative products that meet market demand while preserving the authenticity and cultural features of the museum collections.

Culture can encompass all aspects of human life and the objects created to fulfill them as well as the psychological and behavioral patterns resulting from them [11]. The cultural structure comprises the fixed relationship among various cultural factors within a cultural system, governed by specific rules [12]. Leong and Clark [13] proposed a cultural hierarchy theory that divides cultural space into three levels, namely outer, mid and inner, for analyzing cultural products. The outer “tangible” level focuses on the physical and material aspects of culture, while the mid “behavioral” level includes behavior, institution and traditions. The inner “intangible” level encompasses ideology and non-material spiritual factors. The three levels are considered design features for achieving cultural coherence, and each of them has the potential to influence the impression and satisfaction of the product. Lin [14] added to the three cultural levels by identifying corresponding product design features, such as color and material for the outer “tangible” level; the mid “behavioral” level focuses on product function and operation, and the inner “intangible” level appeals to affection and self-image. The objective of this study was to explore consumer perceptions of the various levels of cultural features found in museum collections. To accomplish this, the study utilized Leong and Clark’s [13] and Lin’s [14] cultural hierarchy theory as a theoretical foundation of extracting and categorizing cultural features.

In order to obtain information about consumers’ attention, purchase intention and satisfaction of museum cultural and creative products, many studies still use traditional information acquisition methods, such as consumer questionnaires [15,16,17,18,19]. These are simple and easy to use, intuitive and straightforward, but they also have the disadvantages of small sample size, high cost, short timeframe and objective bias. By analyzing the online consumption data of museum cultural and creative products, it is possible to overcome the limitations of traditional information gathering methods. In particular, textual reviews reflect the expression of post-purchase emotions of consumers. They also serve as a reference for pre-purchase decisions of potential consumers, so these reviews become an essential information carrier for consumer market analysis. Ali’s e-commerce platform data [20] revealed that, by May 2019, the Tmall store of cultural and creative products of the Palace Museum in Beijing had garnered over 60 million annual visits, exceeding three times the 17 million physical visitors received by the Palace Museum during the same year. This figure significantly surpasses that of other museums, making the “Palace Museum’s Cultural and Creative Flagship Store” the top performer in China’s cultural and creative industry on Tmall.com. Its sales in the festival supplies gift and cultural creation sectors have secured the number one spot, exhibiting an impressive growth rate of nearly double each year. This store’s extensive collection of post-purchase product reviews has provided a comprehensive research dataset on the perceptions of cultural and creative products of museums.

In this context, researching how to accurately extract information on product cultural features, how to analyze consumer perceived needs based on textual reviews and how to evaluate the importance of different cultural feature factors of products are of significant theoretical value and practical significance. Therefore, by utilizing the cultural hierarchy theoretical framework and online text analysis technology, this study establishes a lexicon of features for museum cultural and creative products that can be used to delve into consumers’ perceptions and emotions regarding cultural features. And the results can provide direction for museum designers and managers to purposefully evaluate the cultural features of their collection and their potential applications.

## 2. Research Framework

Drawing upon the three cultural levels of cultural hierarchy theory, this study aims to extract the cultural product features of the Palace Museum. To accomplish this, we employed the research method of text mining and the word2vec model to explore consumers’ perceptions about the cultural features of the museum’s cultural products, using online text review data.

The study comprises four stages. In the first stage, we used a Web crawler tool to retrieve text reviews from e-commerce data and pre-processed them. In the second stage, we invited experts in related fields to extract representative cultural features from the culture of Chinese museum collections using the cultural hierarchy theory. We then selected words or phrases reflecting the meaning of each feature as its seed words to build the seed word set.

In the third stage, we constructed a cultural feature lexicon by processing the Wikipedia corpus and the review corpus using the word2vec word embedding tool based on the seed words. This process generated candidate words and their semantic associations. In the fourth stage, we performed word frequency statistics on the review text and screened the words in the lexicon according to the lexicon of cultural features. We obtained the similarity of the screened words and calculated the product of word frequency and the similarity value of each word. We then calculated the total importance score of each cultural feature by accumulating the scores of all the words successfully matched under it. This helped us to identify the cultural features to which consumers pay more attention.

**Figure 1:**
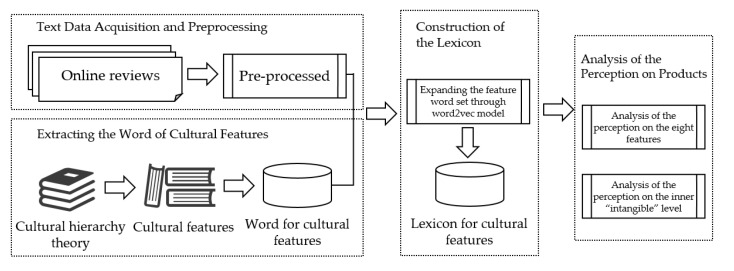
The framework for analyzing consumers’ perceptions based on online reviews and cultural hierarchy theory.

The extraction of high frequency words was completed to better understand consumers’ perceptions of the intrinsic dimensions of the products. The research process is shown in Figure 1.

## 3. Literature Review

### 3.1. Cultural Hierarchy Theory

There are more than 300 definitions of culture worldwide [21]. The British scholar E. B. Tylor was the first to define culture in its entirety, suggesting that culture is a complex whole that includes knowledge, beliefs, arts, morals, laws, customs and all other abilities and habits that people have as members of society. There is a bifurcated approach to the structural anatomy of culture, which is divided into material culture and spiritual culture; a three-level approach, which is divided into material, institutional and spiritual levels; and a four-level approach, which is divided into material, institutional, customs and habits, and ideas and values.

Leong and Clark [13] proposed a framework for incorporating cultural elements into product design. The theory of cultural hierarchy introduces a tripartite model that features three levels of cultural space: outer “tangible” level, mid “behavioral” level and inner “intangible” level. The outer “tangible” level encapsulates the material and tangible aspects of culture, while the mid “behavioral” level refers to the usage behavior and rituals that govern cultural practices. Finally, the inner “intangible” level includes consciousness type and intangible spirit. The three-level structure of the cultural hierarchy framework offers a novel perspective for culturally integrated product research and has been widely embraced in subsequent studies.

Drawing on cognitive psychology, Norman [22] proposed a three-level model of emotional design, which includes the visceral design, behavioral design and reflective design. The visceral design is concerned with the sensory impressions created by the product’s form, material and touch that elicit an initial emotional response. The behavioral design focuses on the pleasure and effectiveness of the product’s use. Finally, the reflective design emphasizes the information conveyed by the product and how it resonates emotionally with users to achieve self-image, personal satisfaction and memories. During product design, designers must consider not only the external visual elements but also the functional behaviors that aid cognition and the emotional effects that relate to users’ internal feelings. The reflective level has the potential to generate sufficient positive emotions to offset or surpass any design deficiencies at the behavioral level. This concept of emotional design can greatly influence the overall success of a product by fostering positive emotional responses from its users [23,24].

**Figure 2:**
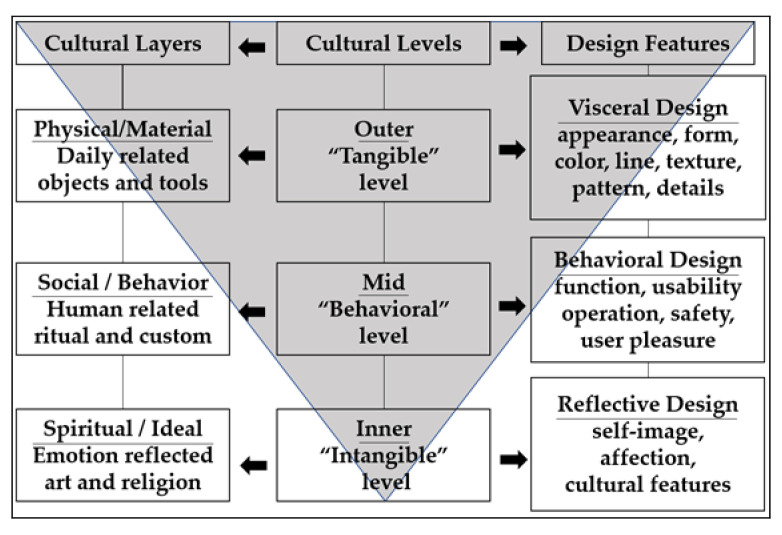
The three layers and levels of cultural objects and design features. Adapted with permission from Ref. [14]. 2007, Rung-Tai Lin.

From the perspective of consumer expectations and cognition, according to Lin [14], the three levels of cultural objects can be correlated with the design features of the three levels proposed by Norman. By merging Leong and Clark’s [13] and Norman’s [22] three-level model, Lin [14] identified the essential features of cultural products that designers must consider during product development. Product design can be approached from three levels of culture. The cultural features are identified in the original cultural object, including those related to the outer level of color, texture and pattern; the mid level of function, usability and safety; and the inner level of emotion, cultural meaning and story-telling. The relationship between cultural levels, layers and design features is illustrated in Figure 2, which can be a useful guide for developing and evaluating cultural products.

These three layers of culture can be fitted into Leong and Clark’s [13] three cultural levels, given above. The design features can be identified when cultural objects are integrated into cultural design.

The outer “tangible” level consists of attributes such as appearance, color, texture, form, decoration, pattern, line quality and details. Visual appeal can improve the perceived value of products for consumers [25]. To create innovative designs for traditional cultural resources, simply transferring traditional images and labels is insufficient. Instead, the design must focus on transforming traditional culture into images that are visually appealing and contemporary [26].

The mid “behavioral” level deals with the function, operational concerns, usability and safety. The traditional functional design approach is “object-centered” and based on rational thinking. It is intended to address practical problems that arise in people’s daily lives and production, with the primary goal of solving specific demands. In contrast, human-centered functional design seeks to shift from being “good to use” to “enjoyable to use.” This approach prioritizes the emotional experience that product functions can provide to users during operation, aligning with human nature’s emotional needs. This emotional experience is essential to creating a positive user experience and can be a crucial factor in determining the overall success of a product. Therefore, designers must consider the emotional experience of their products to create a human-centered functional design that not only meets practical needs but also provides a satisfying emotional experience for users [27].

The inner “intangible” level of product design contains special content, such as stories, emotions and cultural features. Siu [28] suggested that incorporating cultural elements into product design at this level can influence and change people’s decisions and behaviors. The cultural features of the inner “intangible” level can inspire consumers and encourage them to explore the cultural meaning behind the products. For instance, China’s national liquor, Baijiu, is highly prized not only for its exceptional taste (mid-level) but also for its encouragement of the Chinese tradition of combining drinking with artistic expression (inner level) [29]. By incorporating cultural elements into product design, not only can the product acquire a distinct cultural character but it can also strengthen consumers’ emotional connection and cultural identity [30,31]. Chai et al. [32] conducted a refinement and analysis of the elements of cultural symbols in products, developed a feature weighting model and empirically evaluated user satisfaction at various cultural levels. Their findings showed that the inner, intangible cultural elements were more effective in enhancing user satisfaction.

The future of craft design will prioritize humanistic aesthetics, and technology should only serve as a tool for technical support rather than dominating the design process [33]. According to Lin [34], the essence of design lies in its ability to combine art, culture and science to address social issues and redefine human lifestyles. Therefore, he emphasizes the significance of design aesthetics that are based on the principles of “humanity”, “culture” and “community”. Design aesthetics based on the principles of “humanity”, “culture” and “community” prioritize the needs, experiences and values of people in design. This leads to more inclusive, accessible and meaningful products, service and experiences.

This study aims to examine how consumers perceive cultural features in the collections of Palace Museum. To achieve this, the study employs Leong and Clark’s [13] and Lin’s [14] cultural hierarchy theory to extract and categorize cultural features, providing a comprehensive understanding of these elements. It serves as the theoretical foundation for the cultural integration study presented in this study.

### 3.2. Perceptions of Consumption for Museum Cultural and Creative Products

Museum collections embody the essence of traditional culture, and cultural and creative products provide an effective means for museums to promote this rich cultural heritage. By showcasing the cultural significance of their various collections through cultural and creative products, museums can effectively communicate with consumers. Chen and Lei [35] used questionnaires to examine the factors that affect the consumption of cultural and creative products in museums by the younger generation. They discovered that emotional and cultural values have the most significant influence on consumption, with consumers placing a premium on product appearance. Additionally, they found that doll ornaments were highly valued due to their social and emotional significance, which increased the perceived value of the product and lessened consumers’ price sensitivity. Shiau and Hu [2] explored the relationship between the museum’s features and consumer behavior using communication, trust and consumer value as variables. The study found that utilitarianism significantly impacted consumer purchase intentions, suggesting that museums should develop cultural and creative products based on consumers’ perceived utility and economic value. Guo [36] proposed a model and hypothesis on the factors affecting the purchase intention of museum cultural and creative products, based on rational behavior theory and satisfaction theory. The study found that consumers’ perception of the cultural and practical value of such products had a stronger influence on their satisfaction, compared to the cost of consumption.

Chen and Wang [37] conducted a study on the public’s consumption value orientation towards 10 cultural products, including museum cultural products. They analyzed the external form and internal meaning of the products and found that the public’s perception of cultural products was primarily influenced by the shape and color of the products, followed by practicality and overall sense. Combining collection resources with practical products can also increase consumer interest in purchasing as respondents actively inquire about functionality during the questionnaire process. Tu et al. [38] proposed that consumers attach great importance to factors, such as “cultural connotation” and “unique creativity”, when choosing cultural and creative products of the Palace Museum. Among them, men pay more attention to the appearance of the cultural and creative products, while women pay more attention to the connotation of the cultural and creative products. Zhang and Ye [39] classified museum products into three categories: physical, emotional and meaningful. They recommended that adding multisensory experiences can enhance communication, interaction and emotional resonance between users and products.

While previous research has examined cultural hierarchy, cultural product features and the perception of cultural and creative products in museums, our study focuses on two specific areas. First, we utilize the cultural hierarchy theory as a theoretical framework to extract the cultural features of cultural and creative products in the museum and to explore consumers’ perception and experience of these features. Second, we employ text mining techniques and the Word2vec model to analyze online reviews of cultural and creative products from the Palace Museum, which enables us to derive valuable insights from consumers’ review data and texts and to make more objective conclusions.

## 4. Methodology

### 4.1. Sample Websites and Case Selection

The primary reasons for selecting the “Palace Museum’s Cultural and Creative Flagship Store” on Alibaba’s Tmall platform as the empirical study in the analysis of online text evaluations of cultural and creative products in museums are as follows.

First, as a pioneering company in China’s e-commerce industry, Alibaba’s Tmall platform has established itself as a leader in the commercial sector. According to the 2019 Museum’s Cultural and Creative Products Market Data Report, jointly released by Tsinghua University’s Institute of Cultural Economics and Tmall, more than 24 museums have established their online stores on the platform. Of note, the Palace Museum alone has opened six stores on Tmall.com, with the cumulative number of visits to these museum flagship stores reaching 1.6 billion, which significantly exceeds the total annual visits to museums nationwide [40]. Additionally, in 2020, the Palace Museum, National Treasure, Dunhuang Research Institute and National Museum of China were among the top 10 most popular domestic IPs on Tmall. Given the platform’s vast consumer base, it provides a wealth of review information for museum cultural and creative products, thereby making it an ideal source for data collection.

Second, Palace Museum, as a testament to ancient Chinese culture and art, leads the way in the research, development and promotion of cultural and creative products. Presently, the museum operates six online stores on Alibaba: “Palace Museum’s Taobao”, “Palace Museum’s Publishing”, “Palace Museum’s Cultural and Creative Flagship Store”, “Palace Museum’s Food”, “New to the Palace Museum” and “Palace Museum’s Stationery”. Among these, the “Palace Museum’s Cultural and Creative Flagship Store” is particularly noteworthy, having been launched in June 2016 and offering various products, such as decorations, costumes, gifts and more. This flagship store’s practical and elegant style and aesthetic-focused lifestyle concept have endeared it to consumers. Given its considerable impact, diverse product categories and extensive experience in the cultural and creative field, the “Palace Museum’s Cultural and Creative Flagship Store” was selected as the focus of this research.

### 4.2. Text Data Collection and Preprocessing

This study utilized GooSeeker, a web crawling tool, to collect text reviews from consumers who visited the “Palace Museum’s Cultural and Creative Flagship Store” website (https://palacemuseum.tmall.com/ (accessed on 10 May 2022)) on Tmall.com. The data were gathered over a period of five months, from 10 January 2022 to 10 May 2022 and yielded a total of 37,835 reviews. The collected reviews underwent preprocessing, as described below.

First, the natural language processing toolkit Jieba was used to split each line of text into independent words. Second, deactivated words whose meaning could not be identified from the splitting were removed. Finally, the resulting cleaned comment text data was saved as a TXT file, where each line represents a comment composed of multiple separated words. Each comment is denoted as Word (v1, v2, v3, …, vi), with vi being the i-th word in the comment text set in that line. In total, 10,238 valid product review data points were collated in the final compilation.

### 4.3. Identification of the Cultural Features

In studying museum cultural and creative products, identifying their cultural features is crucial. The cultural concept comprises various cultural elements, which have their own specific cultural features [41]. This study focuses on identifying cultural features by synthesizing and summarizing the cultural hierarchy framework presented by Leong and Clark [13] and Lin [14]. The three-level division method of outer “tangible” level, mid “behavioral” level and inner “intangible” level in cultural studies was adopted. The features of each level were defined by combining the characteristics of museum cultural and creative products and the requirements of the study, as outlined in Table 1.

Among the identified features, “Specialty craft” encompasses the production techniques and procedures used in creating cultural and creative products, while “Emotional resonance” refers to the product’s thematic content evoking similar emotional tendencies or responses from consumers. “Aesthetic taste” represents the subjective preferences expressed by the aesthetic subject during the aesthetic process. These features are integral in understanding the cultural aspects of museum cultural and creative products and will aid in the analysis of the collected reviews.

### 4.4. Extraction of Seed Words

To facilitate the analysis of consumers’ perceptions of cultural and creative products, this study employed a research methodology that involved creating a seed lexicon. However, previous studies have failed to provide a comprehensive and inclusive list of seed words required to establish the seed lexicon, given the vast and diverse nature of culture. The seed words proposed in this study may serve as a reference for future research in the field of cultural consumption behavior.

To refine the cultural hierarchy scale for museum cultural and creative products, experts in relevant fields were invited to participate in manual validation. This validation process involved the aggregation of a seed word bank for the feature dimensions, achieved by extracting meaningful seed words and eliminating irrelevant terms. Table 2 outlines the seed words for the cultural features.

### 4.5. Synonym Expansion Based on Wikipedia Corpus

Natural language processing techniques were used in this study to create a comprehensive vocabulary of the museum’s cultural and creative offerings. There are several methods available in the field of natural language processing, including manual annotation, lexicon-based and corpus-based methods. While the manual annotation method yields more accurate results, it is also more time-consuming and labor-intensive. Conversely, the corpus-based method is more flexible for domain-specific applications as it extracts lexical semantic information in real-life contexts, making it a more effective alternative to lexicon-based methods.

Recent advancements in natural language processing have led to the widespread use of word embedding techniques for lexical modeling and correlation analysis among scholars [42]. This method maps words into a high-dimensional vector space, facilitating the discovery of related words with specific meanings in different contexts through inter-vector computation. The success of these methods is largely attributed to their use of large-scale web corpora.

This study utilized the word embedding method to map each vocabulary to a K-dimensional vector space and to calculate the semantic similarity between the words in the corpus and the words in the word set using cosine distance between the vectors. Specifically, a Word2Vec word embedding technique was applied to construct a word vector model using the candidate word set with eight feature dimensions of museum cultural and creative products. This model was then used to identify new words related to cultural and creative themes in a large-scale Internet corpus and review corpus based on lexical similarity.

The cosine similarity is used to calculate the correlation between the word vectors in the candidate lexicon and museum’s cultural and creative products corpus. Make $word_{1}=\left(v_{1}^{w_{1}},v_{2}^{w_{1}},\ldots v_{m}^{w_{1}}\right),\;word_{2}=\left(v_{1}^{w_{2}},v_{2}^{w_{2}},\ldots v_{m}^{w_{2}}\right)$ where m denotes the dimension of the word vector output by the training (set to 200 dimensions), × denotes the dot product between the two vectors. The value of $sim\left(word_{1},word_{2}\right)\;$ranges between −1 and 1, with 1 indicating that the two words are identical and −1 indicating that they are completely opposite.

(1) $$sim\left(word_{1},word_{2}\right)=\cos\theta =\overset{m}{\underset{k=1}{\sum }}\frac{v_{k}^{w_{1}}\times v_{k}^{w_{2}}}{\sqrt{\sum_{k=1}^{m}{\left(v_{k}^{w_{1}}\right)}^{2}\times \sum_{k=1}^{m}{\left(v_{k}^{w_{2}}\right)}^{2}}}$$

In this study, we utilized a method in Gensim software [43] to get the top ten most similar words. Specifically, we employed the Word2vec method and cosine similarity to calculate the correlation between words. To ensure the reliability of our results, we set a similarity threshold of 0.8 based on experimental observations. To obtain the most similar words to the candidate lexicon, we then traversed a word vector model trained from the Wikipedia corpus and reviews corpus. This process was repeated until no new candidate words could be added, resulting in the final extended lexicon. We further improved the accuracy of our findings by retaining only words with a similarity greater than 0.8 to the seed words, while manually removing meaningless words. The resulting cultural feature lexicon represents a comprehensive and accurate understanding of the features associated with museum cultural and creative products.

### 4.6. Calculation of Consumers’ Attention Score for Features

In this study, we calculated the importance score of the cultural features using feature attention scoring, which is a method of scoring input features based on their ability to predict the target object. We followed the below steps to calculate the importance score of cultural features. Firstly, we utilized Python programming to perform word frequency statistics on a total of 10,238 product reviews from the Taobao platform for the Palace Museum’s Cultural and Creative Flagship Store. Next, we filtered words within the lexicon and determined their similarity based on the museum’s cultural and creative product feature lexicon. Words with higher relevance to a specific dimension and higher frequency indicate that consumers attach greater attention to that dimension. Finally, we calculated the attention level of each word under each dimension by combining its word frequency and similarity value. The total attention score of each feature was determined by summing up the product scores of all successful matches under the eight feature dimensions of the museum’s cultural and creative products. We used the following calculation formula to determine the total attention score of each feature:

(2) $$G_{i}=\overset{J}{\underset{j=1}{\sum }}C_{ij}X_{ij}$$

where $G_{i}$ is used to represent the level of consumer attention, while $C_{ij}$ and $X_{ij}$ represent the number of word frequencies and dimension relevance scores of the jth word belonging to dimension i, respectively. Moreover, *J* represents the total number of words belonging to dimension i after filtering. The *G* score reflects the degree of emphasis placed by consumers on each dimension.

## 5. Analysis and Findings

### 5.1. Analysis on the Perception of the Eight Cultural Features

To gauge consumers’ perception levels and the high-frequency word statistics of each dimension of the museum’s cultural and creative product features, the seed word sets of the eight dimensions were matched with the consumer review texts. A higher frequency of the selected dimension’s seed word set in the consumer review text indicates greater concern among consumers. This implies that consumers give more attention to the performance of such product features when purchasing cultural and creative products from museums. Additionally, it serves as the primary reference factor that affects consumers’ purchasing behavior.

The results regarding the quantitative scores of consumers’ perceived attention of the eight features obtained by the feature attention score calculation method are shown in Table 3.

The results of the study indicated that “Materials used” was the most frequently mentioned feature in consumer reviews. Consumers commented on the fabric, sturdiness and roughness of the materials used in the cultural and creative products, such as “the fabric of the socks feels good”, “the material is sturdy and long-lasting”, etc. As the material basis of product design, the quality and workmanship of the materials are crucial for determining the products’ lifespan and are the main concern of consumers. Hence, designers should pay close attention to quality, workmanship and other material aspects.

The study also revealed “External appearance”, “Aesthetic Taste” and “Color” as the next most frequently mentioned features in consumer reviews. These features, particularly “External appearance” and “Color scheme”, reflect the features of the outer “tangible” level. This highlights that the external design of museum cultural and creative products is highly recognizable to consumers and plays a significant role in shaping their consumption behavior. The perception of a product’s shape and color will directly impact people’s evaluation of it. “Aesthetic taste” encompasses social, contemporary and national aspects, and the high level of consumer focus on this feature highlights the significance of comprehending current consumer preferences and being mindful of the delicate balance between traditional cultural sensitivities and individual aesthetics for attaining commercial success.

The features of “Functional feature”, “Stories and legends”, “Emotional resonance” and “Characteristic craftsmanship” received less attention. Based on the findings, it can be inferred that the three cultural levels of museum creative products are from the outside to the inside, and the more implicit the cultural elements are, the more difficult they are to be recognized by consumers.

### 5.2. Analysis on the Perception of Inner “Intangible” Level

The analysis of high-frequency words involved in the three dimensions of “Stories and legends”, “Emotional resonance” and “Aesthetic taste” at the inner “intangible” level further delves into the consumers’ cultural perception tendencies at this level. The results of the high-frequency word classification are presented in detail in Table 4.

Culture is the value guardian of old traditions, and cultural creation fuels new life; however, its core remains rooted in cultural values [44]. The sustainability of museum cultural and creative products is evaluated not only by the economic returns they generate but also by their impact on promoting the cultural heritage of their collections. The findings suggest that consumers’ perceptions of the Palace Museum’s cultural and creative products primarily revolve around the “Palace”, with broader terms like “story” and “traditional culture” being frequently used. Conversely, more specific terms like “Riverside Scene at Qingming Festival” and “hero” are mentioned less often in online reviews, indicating a lack of awareness regarding the cultural significance of the museum’s resources.

Consumer behavior is driven by emotions. The demand for museum cultural and creative products is not only driven by the functional and aesthetic aspects but also by the emotional appeals and expressions of desire [22,45,46]. When it comes to “Emotional resonance”, consumers frequently express their emotional connection to the museum’s products through words such as “joyful”, “trust”, “satisfied” and other positive terms, indicating that the museum’s cultural and creative products make consumers feel the infectious power of the products radiating out, causing certain positive resonance. The high-frequency words related to “Aesthetic taste” are mainly positive, including “beautiful”, “exquisite”, “novelty”, “lovely”, “dignified”, etc. In general, Palace Museum’s cultural and creative products are designed to align with mainstream aesthetic style and follow the central trends in human social and cultural developments.

## 6. Conclusions and Discussion

This study takes the Palace Museum’s Cultural and Creative Flagship Store as a case study and uses Word2vec data analysis technology to analyze online textual reviews of the museum’s cultural and creative products. The findings of this study shed light on consumers’ perceptions of the cultural features of these products and deepen our understanding of consumers’ cultural needs.

The cultural features that consumers pay the most attention to in the Palace Museum’s cultural and creative products is “Material used”, followed by “External appearance”, “Aesthetic taste”, “Color scheme”, “Functional feature”, “Stories and legends” and “Emotional resonance.” In contrast, “Specialty craft” receives the least attention. Consumers place greater emphasis on the outer aspects of these products, reinforcing the findings of previous studies [35,37]. Furthermore, consumers’ preferences for product appearance vary widely. For example, one creative product, the “coffee cup”, received comments such as “the actual color is a little darker”, while others stated “the cup is very beautiful, the pattern is clear and colorful”, demonstrating contrasting opinions.

Most cultural feature factors at the mid “behavioral” level and inner “intangible” level were not effectively identified in our study. Specifically, the perception of cultural backstory and function of museum cultural and creative products was found to be insignificant. The lack of clarity in the product development’s cultural background information is a potential explanation for the low perception of the inner “intangible” level among consumers. Additionally, many of these cultural and creative products are indistinguishable from ordinary souvenirs and have limited application scenarios, leading to a lower perceived practical value among consumers. The market is also saturated with similar cultural and creative products, making it challenging to differentiate and showcase unique craftsmanship.

This study analyzed the inner “intangible” cultural perception tendencies of consumers towards the Palace Museum’s cultural products by examining three cultural features: “Stories and legends”, “Emotional resonance” and “Aesthetic taste.” The results suggest that consumers generally appreciate the cultural expression inherent in the museum’s cultural and creative products. However, the study also found that the background knowledge of the products was rarely mentioned in the texts, indicating a lack of effective content and storytelling in the innovative products.

Interestingly, despite the fact that many consumers may not be familiar with the historical significance of the Palace, the sales figures of Forbidden City cultural merchandise remain impressive. Additionally, the frequent use of the word “Palace” in post-purchase reviews suggests that consumers are attracted to these products partly because of the name. This underscores the Palace’s influential branding impact and the effectiveness of cultivating cultural symbols for the brand.

## 7. Suggestions

According to the study results, development strategies for museum cultural and creative products can be started from the following points.

Consumers are especially discerning about the materials used and often prioritize them as the most important factor. Despite the “commemorative” nature of museum products, their expectations for materials remain high and may even be higher compared to non-museum products. Designing museum cultural and creative products with unique materials can increase their differentiation and challenge consumers’ stereotypical views about such products and their materials. Additionally, while maintaining product reliability, it is important to minimize material and energy consumption and to utilize technology to explore new forms and facets of materials. This can result in new and engaging aesthetic experiences that captivate the consumer’s attention.

The external appearance and color of museum cultural and creative products are crucial to consumer perception and belong to the outer cultural level. Research has shown that visual art in product design, advertising and packaging can have a positive impact on consumer evaluation [47]. In order to effectively develop museum cultural and creative products, it is important to pay close attention to the appearance and shape of the products and adhere to design aesthetics principles. Consumers generally prefer a harmonious, balanced and coordinated appearance [48]. Before launching these products, conducting market research to understand consumer preferences and adapting product design accordingly is crucial. This research can involve analyzing data on consumer classification, color preferences, brand trends and transaction data to evaluate the value of product appearance and to personalize the design. In addition, it is important to maintain consistency in terms of color, shape and details to achieve a commercial, fashionable, simple and elegant aesthetic.

The development of museum cultural and creative products should respect the cultural essence they embody, prevent the overuse of cultural elements and steer clear of superficial formalism. To do so, museums must delve into the cultural significance and historical context of their collections, which will allow them to create added value through artistic expression. By combining traditional cultural elements with new materials and technologies, museums can create unique cultural symbols, better communicate their cultural significance to consumers and establish a three-dimensional brand image in an increasingly homogenized cultural and creative market. As an example, Taipei Palace Museum and Shang Tang Technology have jointly released a digital cultural product, “Squabble in the Mountains—Three Great Song Paintings”, which brings a three-dimensional world of Song Dynasty Xi Shan to life through physical and digital elements. Similarly, Suzhou Museum has created a series of cultural and creative products, such as “Tang Yin Tea Bag”, inspired by the Intellectual Property of Tang Bohu and other characters from south of the Yangtze River. These products allow for deeper cultural connotations of the Ming dynasty to be more accessible to consumers and enhance the museum’s brand and cultural image, while also generating economic benefits.

A strong emotional bond between the product and the user is key to the success of these products [49]. As designer Kenya Hara once noted, they should “rediscover the senses”. According to modern product design, they should focus on serving the real-life needs of individuals, with daily life serving as their spiritual foundation [50]. “Cultural Creation Ice Cream” is a prime example of how museums can merge cultural relics and food, infusing new life into traditional products and providing a full sensory experience for consumers. The development of museum cultural and creative products should bring the cultural significance of the collection to life through the products, broadening their use and making culture more accessible, tangible and usable. This requires encouraging communication between people and objects, triggering new cultural insights and creating empathy with the products. By doing so, cultural content is effectively shared, and the products are promoted, resulting in the rapid growth of the cultural and creative brand and market.

To expand the breadth and depth of dissemination, museums can launch offline exhibition models in addition to selling products online. Rare cultural relics in museums are presented as digital short films to deepen consumers’ understanding of the semantic meaning of cultural and creative products. By linking the dual platforms of offline experience and online purchase, cultural communication can extend its reach and achieve multidimensional promotion. In addition, museums need to fully utilize their educational function by designing usage scenarios for cultural and creative products based on their connotations, taking advantage of the relationship between young people’s characteristics, events, products and environment and combining them with areas of interest. For instance, major museums have launched a range of cultural and creative products and experience projects that feature “game+” elements. These include the interactive puzzle game book series “Mystery Palace” by the Palace Museum, the “Lost Treasures” blind box series by the Henan Museum and the painted “Shixi” by the Chengdu Museum, which enhances the value proposition and educational value of museums [51]. A positive feedback loop of mutual promotion has been formed between museum cultural and creative products and consumers, with stable consumer market order bringing better social benefits and promoting sustainable product development.

## 8. Limitations and Future Research

This study has some limitations that need improvement in the future. First, the study did not select data based on reviews of cultural and creative products from other e-commerce websites, and the experimental subjects’ data size and categories need to be expanded. Consumers who use different websites to purchase cultural and creative products may have group differences, such as gender and age, which may affect the importance of the perception dimension. Second, this study constructs seed words from a cultural theoretical level. However, since there are many and varied words describing cultural resources, the word set may not be complete. Therefore, the lexicon needs to be updated for subsequent research. Third, due to the short content, obvious colloquialisms and unclear meaning of sentences in textual reviews of e-commerce platforms, the method used in this study cannot identify such statements. Some scholars extract product features by analyzing the correlation and dissimilarity of the two corpora to improve the accuracy of feature extraction [52]. Future research can be improved according to this idea. In addition, there are other challenges associated with online reviews, such as limited data from older folks and susceptibility to bias and false reviews. These obstacles can hinder researchers from obtaining precise and valuable insights. As a solution, a combination of online reviews and questionnaires can be used in future research exploring the consumption behavior of museum cultural and creative products.

## Figures and Tables

**Table 1:** Features of cultural and creative products.

Cultural Levels	Cultural Features
Outer “tangible” level	Color scheme
	External appearance
	Materials used
Mid “behavioral” level	Specialty craft
	Functional feature
Inner “intangible” level	Stories and legends
	Emotional resonance
	Aesthetic taste

**Table 2:** Seed words for cultural features.

Cultural levels	Cultural Features	Seed Words
Outer “tangible” level	Color scheme	Color, paint, monochrome, mixed colors, chromatic, bright, color match, dark, red, vivid, dull
	External appearance	Shape, pattern, size, motif, structure, decoration, round, regular, distort, appearance
	Materials used	Material, quality, texture, touch, cotton, wood, thick, rough, smooth
Mid “behavioral” level	Specialty craft	Working process, handwork, craft, carving, embroidery, patchwork, cutting, hollowing, knotting, fire-starting, artistry, technology, thickening
	Functional feature	Function, lighting, education, commemoration, decoration, storage, protection, reading, collection, operation, drinking, viewing, usability, gift, safety
Inner “intangible” level	Stories and legends	Legend, dynasty, era, story, festival, Jianghu, emperor, annals, connotation, deities, symbolism, narrated, traditional culture, Forbidden City, custom, folk custom
	Emotional resonance	Sentiment, feeling, joy, trust, fear, surprise, sadness, disgust, anger, anticipation, love, emotion
	Aesthetic taste	Aesthetic, taste, novelty, elegance, adorable, trendy, fresh, old-fashioned, dorky, casual, ordinary, tacky

**Table 3:** Total attention score of cultural features.

Cultural Levels	Cultural Features	Total Score	Percentage (%)	Ranking
Outer “tangible” level	Color scheme	8308.459	14.47%	4
	External appearance	9973.430	17.37%	2
	Materials used	10,592.895	18.44%	1
Mid “behavioral” level	Specialty craft	3268.986	5.69%	8
	Functional feature	5908.964	10.29%	5
Inner “intangible” level	Stories and legends	5693.308	9.91%	6
	Emotional resonance	3985.595	6.94%	7
	Aesthetic taste	9702.962	16.89%	3

**Table 4:** Classification of high-frequency words.

Cultural Levels	Cultural Features	High-Frequency Words (Frequency)
Inner “intangible” level	Stories and legends	Palace (2675) story (416) traditional culture (346) Jiangshan (276) immortal (169) Riverside Scene at Qingming Festival (70) hero (40)
	Emotional resonance	joy (594) trust (415) satisfied (155) attentive (111) deflated (90) embarrassed (52)
	Aesthetic taste	beautiful (3692) exquisite (1025) novelty (568) lovely (402) dignified (299) ugly (143) classy (119)

## Data Availability

The data presented in this study are available from the corresponding author upon request.
